# Factors associated with mortality after proximal femoral fracture

**DOI:** 10.1186/s10195-023-00715-5

**Published:** 2023-06-26

**Authors:** Nike Walter, Dominik Szymski, Steve Kurtz, Volker Alt, David W. Lowenberg, Edmund Lau, Markus Rupp

**Affiliations:** 1 Department of Trauma Surgery, University Medical Center Regensburg, Franz-Josef-Strauß-Allee 11, 93053 Regensburg, Germany; 2 Department of Psychosomatic Medicine, University Medical Center Regensburg, Regensburg, Germany; 3 Implant Research Core, School of Biomedical Engineering, Science and Health Systems, Drexel University, Philadelphia, USA; 4 Department of Orthopaedic Surgery, Stanford School of Medicine, Stanford, USA; 5 Exponent Inc., Palo Alto, USA

**Keywords:** Proximal femur fractures, Mortality rate, Risk factors

## Abstract

Proximal femoral fractures are a serious complication, especially for elderly patients. Therefore, we have aimed to answer the following research question: What is the postfracture mortality rate in the elderly population and what are associated risk factors? For this, proximal femoral fractures that occurred between 1 January 2009 and 31 December 2019 were identified from the Medicare Physician Service Records database. The Kaplan–Meier (KM) method with the Fine and Gray subdistribution adaptation was used to determine rates of mortality. A semiparametric Cox regression model was applied, incorporating 23 measures as covariates to identify risk factors. The estimated 1 year mortality rate was 26.8% after head/neck fracture, 28.2% after intertrochanteric fracture, and 24.2% after subtrochanteric fracture. Male sex, age over 70 years, chronic obstructive pulmonary disease (COPD), cerebrovascular disease, chronic kidney disease, a concomitant fracture, congestive heart failure, diabetes mellitus, hypertension, insulin use, ischemic heart disease, morbid obesity, osteoporosis, tobacco dependence, and median household income were determined as risk factors for increased mortality. An early assessment of individual risk factors accessible for therapeutic treatment is crucial in the management of proximal femur fractures to aid in attempts at reducing the high mortality apparent in the elderly US population.

## Introduction

Proximal femoral fractures are one of the most common fractures. Projections anticipate a prevalence of up to 21.3 million annually cases worldwide in the year 2050 [1]. In particularly for elderly patients, these types of fracture are associated with severe complications. The aim of our study was to investigate the mortality rate and influencing factors for all types of proximal femoral fractures.

## Methods

Proximal femur fractures that occurred between 1 January 2009 and 31 December 2019 in patients ≥ 65 years were identified from the Medicare Physician Service Records database. The 5% sample of Medicare beneficiaries, equivalent to the records from approximately 2.5 million enrollees formed the basis of this study. The International Classification of Diseases Ninth and Tenth Revisions, were used to identify femur fractures from these physician records. Fractures were grouped into head/neck, intertrochanteric, and subtrochanteric fractures. Postfracture mortality risk was investigated as the outcome. Mortality was identified from the Medicare enrollment data, provided by the Centers for Medicare and Medicaid Services. For mortality comparison, Medicare enrollees with no femur fracture but of the same 5 year age group, sex, and resident county area was used. The Kaplan–Meier (KM) method with the Fine and Gray subdistribution adaptation was used to calculate mortality rates. We also used semiparametric Cox regression with competing risk correction to investigate risk factors. The Cox models incorporated demographic, clinical, and several community-level socioeconomic measures as covariates. All data processing and statistical analyses were performed using SAS statistical software (version 9.4, Cary, NC) and significance was determined at α = 0.05.

## Results

**Fig. 1:**
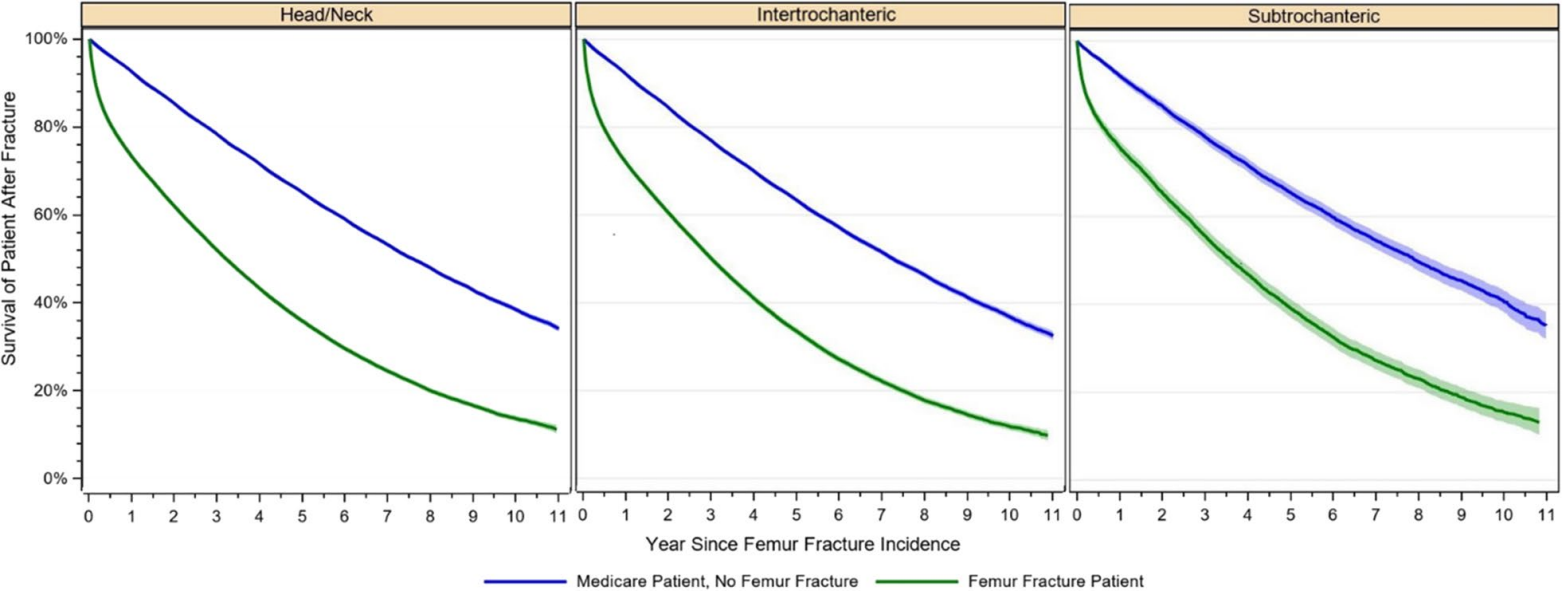
Survival of patients after proximal femur fracture in comparison with other Medicare enrollees without femur fracture

After 1 year, 73.2% (95% CI 72.7–73.6%) of head/neck fracture patients, 71.8% (95% CI 71.1–72.4%) of the intertrochanteric fracture patients, and 75.8% (95% CI 74.1–77.3%) of the subtrochanteric fracture patients survived, whereas after 11 years, 11.2% (95% CI 10.1–12.3%) of head/neck fracture patients, 9.8% (95% CI 8.6–11.0%) of the intertrochanteric fracture patients, and 13.2% (95% CI 10.2–16.5%) of the subtrochanteric fracture patients were still alive. In comparison with Medicare patients without a femur fracture, a mean survival difference of −26.09% was observed for head/neck fracture cases. For intertrochanteric fracture patients the difference was −26.6% on average and 2–4.6% for subtrochanteric fractures (Fig. 1).

The mortality risk was higher after intertrochanteric fractures compared with head/neck fracture (HR = 1.04, 95% CI 1.03–1.05, *p* < 0.001) as well as subtrochanteric fractures (HR = 1.12, 95% CI 1.09–1.15, *p* < 0.001). Mortality risk was lower in women than in men (HR = 0.73, 95% CI 0.72–0.84, χ^2^ = 1059.4, *p* < 0.001). Further, risk of death was increased in patients aged 70–74 years (HR = 1.08, 95% CI 1.03–1.14, χ^2^ = 10.26, *p* < 0.001), in patients aged 75–79 years (HR = 1.45, 95% CI 1.38–1.52, χ^2^ = 242.14, *p* < 0.001), and in patients older than 80 years (HR = 2.65, 95% CI 2.54–2.77, χ^2^ = 2078.33, *p* < 0.001) compared with patients aged 65–69 years. Other significant risk factors are summarized in Table 1.

**Table 1:** Multivariate analysis of mortality risk factors after proximal femur fractures

*Factor*	*HR*	*Lower HR*	*Upper HR*	*Chi-square*	*p*-Value
Age 70–74 years	1.08	1.03	1.14	10.26	0.001
Age 75–79 years	1.45	1.38	1.52	242.14	< .001
Age 80+ years	2.65	2.54	2.77	2078.33	< .001
Female sex	0.73	0.72	0.74	1059.43	< .001
Anticoagulant use	0.99	0.96	1.02	0.37	0.542
COPD	1.45	1.42	1.48	1074.94	< .001
Cerebrovascular disease	1.18	1.15	1.21	198.53	< .001
Chronic kidney disease	1.29	1.26	1.33	397.18	< .001
Concomitant fracture	1.05	1.03	1.08	15.22	< .001
Congestive heart failure	1.54	1.51	1.58	1294.55	< .001
Diabetes mellitus	1.05	1.03	1.07	19.93	< .001
Fall-related fracture	0.99	0.97	1.00	1.94	0.164
Hypertensive disease	0.94	0.92	0.96	39.07	< .001
Insulin use	1.22	1.12	1.34	19.98	< .001
Ischemic heart disease	1.03	1.01	1.05	7.63	0.006
Morbid obesity	0.84	0.74	0.96	6.91	0.009
Open fracture	1.00	0.94	1.05	0.03	0.869
Osteoporosis	0.84	0.82	0.86	161.93	< .001
Rheumatoid disease	1.01	0.96	1.07	0.28	0.594
Tobacco dependence	1.14	1.08	1.22	18.15	< .001
College degree (%)	1.00	1.00	1.00	0.82	0.364
Poverty (%)	1.00	0.99	1.00	3.16	0.076
Unemployed (%)	0.99	0.98	0.99	14.62	< .001
Median income	0.99	0.98	0.99	22.43	< .001

*HR* hazard ratio

## Discussion

The estimated 1 year mortality rate of 26.8% after head/neck fracture, 28.2% after intertrochanteric fracture, and 24.2% after subtrochanteric fracture is consistent with the literature, with others reporting rates of 21.2%, 23.0%, 20.7%, and 18.8%, respectively, for proximal femur fractures in the elderly [2–5]. Male sex, age over 70 years, chronic obstructive pulmonary disease (COPD), cerebrovascular disease, chronic kidney disease, a concomitant fracture, congestive heart failure, diabetes mellitus, hypertension, insulin use, ischemic heart disease, morbid obesity, osteoporosis, tobacco dependence, and median household income were determined as risk factors for increased mortality. Other investigations confirmed these results and also reported a higher mortality risk associated with a low Parker mobility score (OR = 2.94, 95% CI 1.31–6.57, *p* = 0.01), a Charlson-Comorbidity score of 4 or greater (OR = 2.15, 95% CI   1.30–3.55, *p* = 0.002) [2], as well as in patients affected by more than two comorbidities (respectively OR_30 day_ = 2.003, OR_6 month_ = 1.8654, and OR_1 year_ = 1.5965) [5]. In a meta-analysis of 18 cohort studies published by Liu et al. (2018), the majority of identified risk factors were verified, while in this analysis male sex (HR 1.91, *p* < 0.001) rather than female sex was reported as an influencing factor. Surprisingly, the meta-analysis did not show an increased mortality risk for diabetes mellitus (HR 1.15, *p* < 0.121) or nicotine consumption (HR 1.54, *p* < 0.337) [6].

## Conclusion

In this case–control study, a high mortality for proximal femur fractures was apparent in the elderly US population. An early assessment of individual risk factors accessible for therapeutic treatment is crucial in the management of proximal femur fractures to aid in attempts at reducing mortality.

## Data Availability

The datasets used and/or analyzed during the current study available from the corresponding author on reasonable request.
